# Identifying Suitable Locations for Mesophotic Hard Corals Offshore of Maui, Hawai‘i

**DOI:** 10.1371/journal.pone.0130285

**Published:** 2015-07-08

**Authors:** Bryan Costa, Matthew S. Kendall, Frank A. Parrish, John Rooney, Raymond C. Boland, Malia Chow, Joey Lecky, Anthony Montgomery, Heather Spalding

**Affiliations:** 1 National Centers for Coastal Ocean Science Biogeography Branch, National Oceanic and Atmospheric Administration, Silver Spring, Maryland, United States of America; 2 CSS-Dynamac, Fairfax, Virginia, United States of America; 3 Pacific Islands Fisheries Science Center, National Oceanic and Atmospheric Administration, Honolulu, Hawai‘i, United States of America; 4 Office of National Marine Sanctuaries, National Oceanic and Atmospheric Administration, Honolulu, Hawai‘i, United States of America; 5 Pacific Islands Fish and Wildlife Office, United States Fish and Wildlife Service, Honolulu, Hawai‘i, United States of America; 6 Deparment of Botany, University of Hawai’i at Mānoa, Honolulu, Hawai‘i, United States of America; University of Glasgow, UNITED KINGDOM

## Abstract

Mesophotic hard corals (MHC) are increasingly threatened by a growing number of anthropogenic stressors, including impacts from fishing, land-based sources of pollution, and ocean acidification. However, little is known about their geographic distributions (particularly around the Pacific islands) because it is logistically challenging and expensive to gather data in the 30 to 150 meter depth range where these organisms typically live. The goal of this study was to begin to fill this knowledge gap by modelling and predicting the spatial distribution of three genera of mesophotic hard corals offshore of Maui in the Main Hawaiian Islands. Maximum Entropy modeling software was used to create separate maps of predicted probability of occurrence and uncertainty for: (1) *Leptoseris*, (2) *Montipora*, and (3) *Porites*. Genera prevalence was derived from the in situ presence/absence data, and used to convert relative habitat suitability to probability of occurrence values. Approximately 1,300 georeferenced records of the occurrence of MHC, and 34 environmental predictors were used to train the model ensembles. Receiver Operating Characteristic (ROC) Area Under the Curve (AUC) values were between 0.89 and 0.97, indicating excellent overall model performance. Mean uncertainty and mean absolute error for the spatial predictions ranged from 0.006% to 0.05% and 3.73% to 17.6%, respectively. Depth, distance from shore, euphotic depth (mean and standard deviation) and sea surface temperature (mean and standard deviation) were identified as the six most influential predictor variables for partitioning habitats among the three genera. MHC were concentrated between Hanaka‘ō‘ō and Papawai Points offshore of western Maui most likely because this area hosts warmer, clearer and calmer water conditions almost year round. While these predictions helped to fill some knowledge gaps offshore of Maui, many information gaps remain in the Hawaiian Archipelago and Pacific Islands. This approach may be used to identify other potentially suitable areas for MHCs, helping scientists and resource managers prioritize sites, and focus their limited resources on areas that may be of higher scientific or conservation value.

## Introduction

Predictive modelling plays an important role in the study of organisms that are logistically difficult to sample broadly. Such models help researchers and managers explore questions related to spatial ecology and place-based conservation in the absence of timely, *in situ* information about these organisms. They can also help raise new questions about an organism’s ecology by identifying complex relationships in their environment [1]. While predictive models cannot replace the collection of *in situ* information, they can help scientists target data collection efforts to answer specific biological or ecological questions, and help them identify new locations for future scientific research and exploration.

Mesophotic coral ecosystems (MCEs) and mesophotic hard corals (MHCs) are examples of such ecosystems and organisms that are hard for human divers to access because they live in depths (ca. 30 to 150 m) largely beyond recreational SCUBA limits (ca. 40 m) [2]. Consequently, the vast majority of coral reef studies have been conducted on shallow reef systems including those in the Main Hawaiian Islands (MHI) [3]. The limited studies on MCEs have revealed some biological and ecological connections to shallow reefs [3, 4, 5, 6, 7], but much is still unknown about the overall degree of connectivity [6]. This critical information gap must be filled to help managers better understand the role of MCEs in coral reef resilience and in comprehensive conservation strategies to promote healthy and resilient coastal ecosystems and communities.

Although upper MCEs (≤60 m) typically include hard coral species generally found in shallow-water reefs [6], lower MCEs (>60 m) are often biologically distinct from their shallow-water counterparts because they host species of hard corals that are not found in shallower waters [4, 6, 8, 9]. These different hard coral species show a range of adaptations which allow them to live in low light environments, including flattened morphologies, pigment specialization, increased heterotrophy, and lower metabolic demands [8]. In the Hawaiian archipelago, Kahng and Kelley (2007 [9] and Rooney et al. (2010) [10] found that different species and genera of MHCs dominated different depth ranges. Notably, in 50 to 75 m depths, MCEs were dominated by dense stands of Montipora with low-relief branching and plate-like morphologies; in 75 to 130 m depths, MCEs were dominated by *Leptoseris* species [7] with plate-like to foliaceous morphologies. Several environmental factors are thought to help explain this vertical habitat partitioning, including differences among water currents, water temperature, amount of uncolonized hard substrate and the availability of photosynthetically active radiation (PAR) at depth [8, 9, 11, 12, 13, 14].

However, it remains unknown how these (and other environmental variables) interact to constrain MCE and MHC distributions across horizontal and vertical space. To help begin to fill this knowledge gap, we compiled data describing 34 environmental factors thought to be important for identifying areas suitable for MCEs and MHCs. Maximum Entropy (MaxEnt) modeling software [15] was used to identify the combination of key environmental drivers explaining the spatial distributions of three MHC genera (i.e., *Leptoseris*, *Montipora* and *Porites*) offshore of Maui in the MHI [16]. We chose to focus on *Leptoseris*, *Montipora* and *Porites* because they are the three most common MHC genera offshore of Maui. MaxEnt was also used develop spatial predictions of probability of occurrence for each genus (using genera prevalence derived from *in situ* presence/absence data). The models and spatial predictions provide insight into the environmental conditions driving MHC distributions.

## Methods

### Study Area

**Fig 1:**
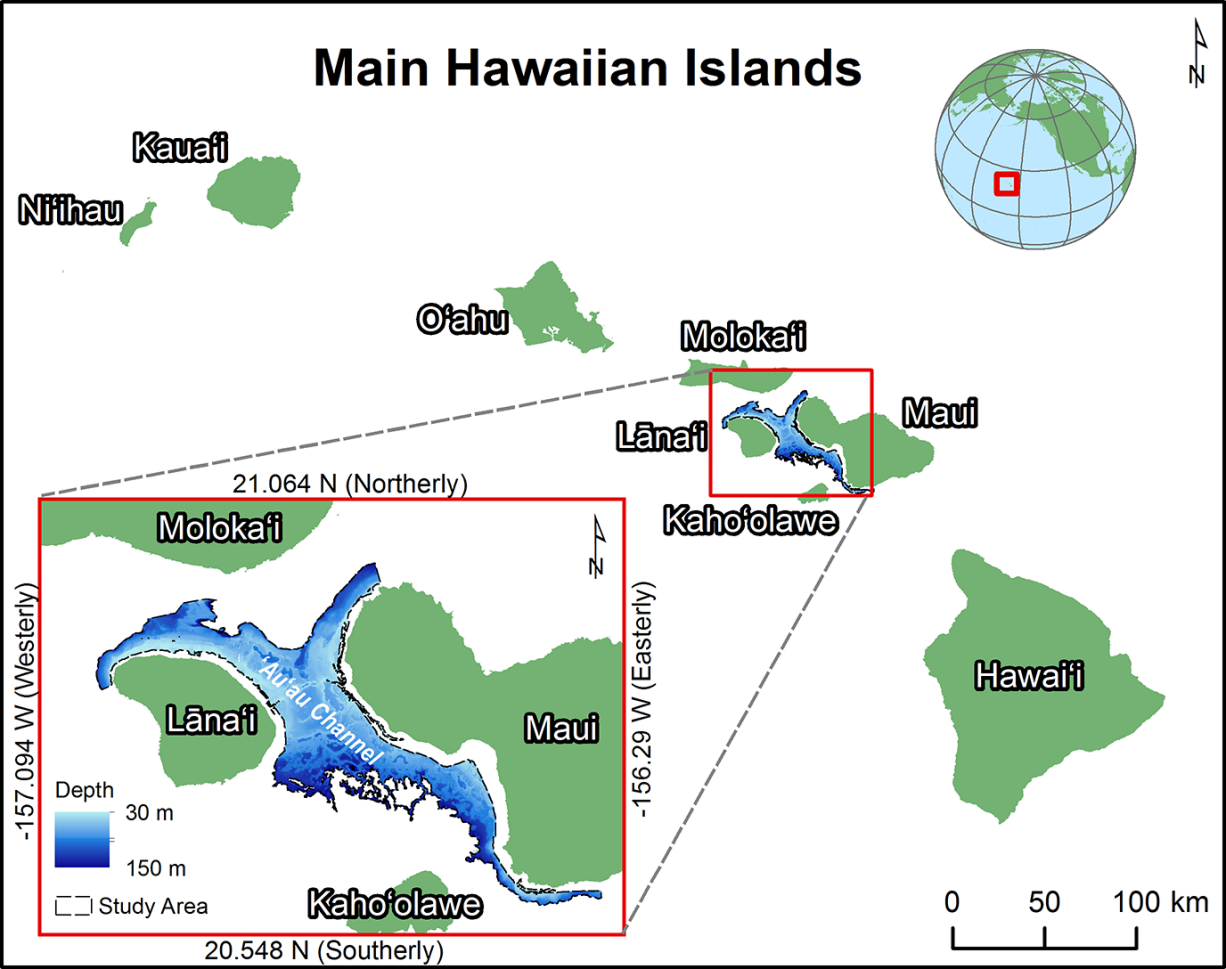
Study area. This map shows the study site offshore of Maui, Hawai‘i.

**Fig 2:**
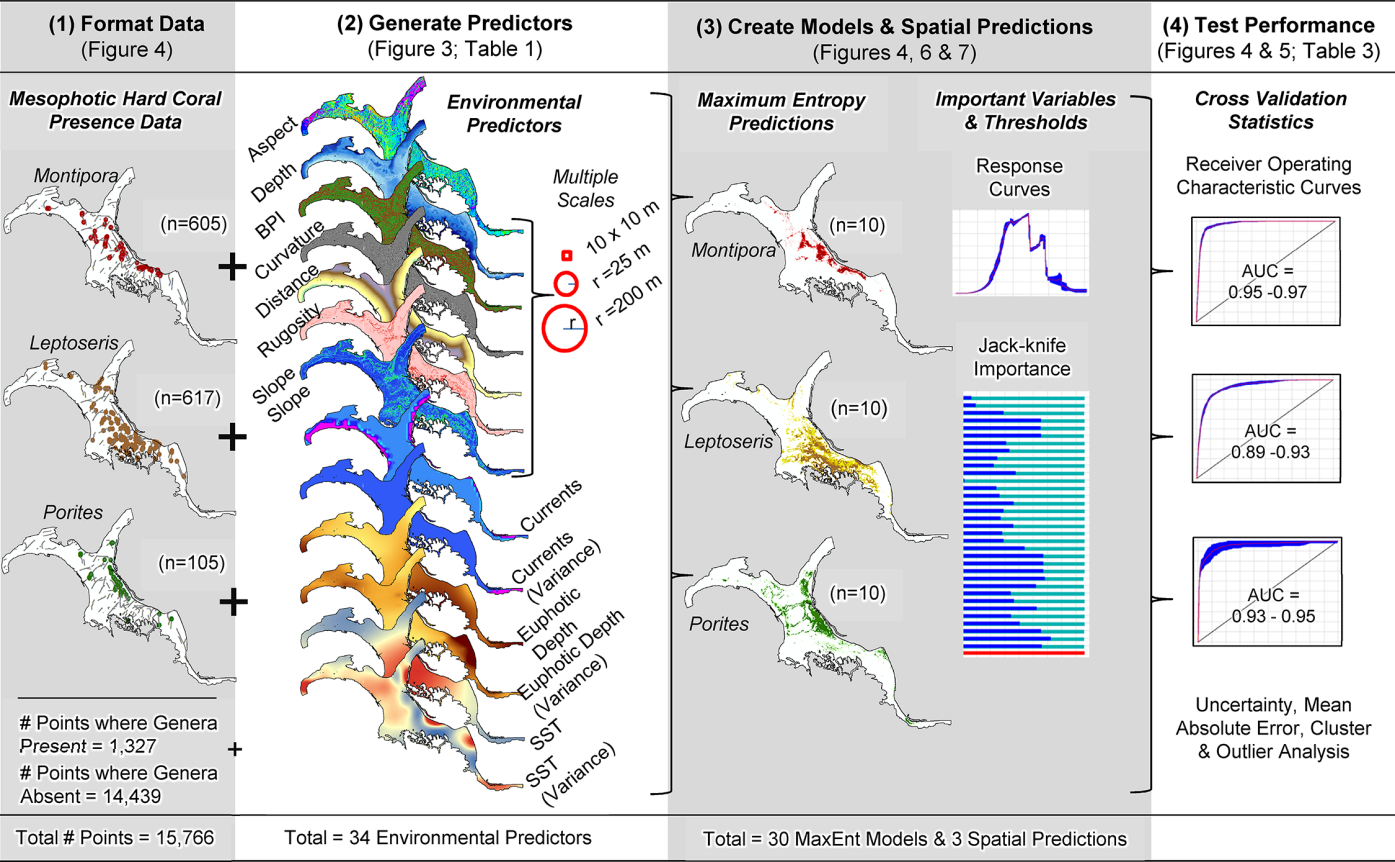
Diagram of modeling process. Steps used to develop MaxEnt models and spatial predictions for *Montipora*, *Leptoseris* and *Porites*.

The focus of this study was a 30 to 150 m deep area between the islands of Maui, Lāna‘i, Moloka‘i, and Kaho‘olawe, including the ‘Au‘au Channel (Fig 1). Fig 2 shows an overview of the process used to predict the locations of MHCs in this study area. This study area was chosen because it lies within the boundaries of a marine protected area, which is considering expanding its scope to include conserving and managing MCEs. It also encompassed the majority of georeferenced information about the presence and absence of MHCs. Several physical conditions are thought to make the study area an ideal place for MHCs including having consistently good water quality and clarity because it: (1) is flushed by tidal currents semi-diurnally [17]; (2) has lower amounts of rainfall and sediment run-off from the nearby land [18]; and (3) is protected from seasonally strong wind and wave energy [19]. Combined, these weather and oceanographic conditions create patches of comparatively warm, calm, and clear waters that remain relatively stable through time.

### Mapping the Locations of Mesophotic Hard Corals

The locations of MHCs were compiled from underwater video and photos collected in the study area during fourteen research missions from November 25, 2001 to September 26, 2011. The field surveys were conducted in State of Hawaii waters, and no specific permissions were required (Hawaii, Department of Land and Natural Resources HAR 13–91 and 13–95). The surveys did not involve endangered or protected species. The bounding coordinates for the area surveyed are: top = 21.064 N, bottom = 20.548 N, right = -156.29 W, left = -157.094 W. A Towed Optical Assessment Device (TOAD) camera sled was deployed in 2008 to 2011 and a RCV-150 Remotely Operated Vehicle (ROV) was deployed in 2001, 2002, 2004 and 2006 to 2011 [8, 20]. The positional uncertainty associated with the TOAD and ROV videos range from ± 15 to 100 m. Video data from these fourteen research missions were classified every 30 seconds at 5 points spaced equidistantly in a horizontal line across the monitor screen [8, 20]. Substrate type, living biological cover (including hard coral, crustose coralline algae, macroalgae and soft corals) and other benthic characteristics were recorded in the classification process. The resulting 17,282 georeferenced records were merged into a single shapefile, and projected into a common coordinate system (i.e., NAD 83 UTM 4 N). We removed 1,516 points because they were unclassified (n = 1,443) or denoted the locations of corals other than *Montipora*, *Leptoseris* and *Porites* (n = 73). These deletions left 15,766 points in total, of which 14,439 points identified locations where *Montipora*, *Leptoseris* and *Porites* were absent. The remaining 1,327 points denoting presence were used to develop genus-specific coral distribution models for *Montipora*, *Leptoseris* and *Porites*. These points were collected between 2004 and 2010.

### Mapping the Environmental Conditions

Several physical factors are thought to influence MHC distributions, including water temperature (at depth), currents (at depth), hard substrate complexity and availability, water chemistry (i.e., pH, aragonite saturation, alkalinity), and the availability of PAR (at depth) due to turbidity and cloud cover [8, 10, 20, 21, 22, 23, 24, 25]. Given their suspected importance, many of these physical factors were included in this modeling effort as predictor variables. Table 1 describes each of the predictor variables included in this study. Information about water chemistry, water temperature (at depth) and variation in cloud cover were excluded because data for these variables were not readily available for the study area or the time period (2004 to 2010) when the 1,327 points used to develop MaxEnt models were collected. Measures of seafloor topographic complexity were used as a proxy for the availability of hardbottom, since a comprehensive map of hard and soft seafloor sediments was also not readily available.

**Table 1:** Descriptions of environmental predictors. This table describes the environmental predictors used to develop the MaxEnt models for *Montipora, Leptoseris* and *Porites*. These predictors were compiled from a variety of sources, including the National Oceanic and Atmospheric Administration (NOAA), University of Hawai‘i, Bishop Museum, Hawai‘i Department of Aquatic Resources (DAR), U.S. Fish and Wildlife Service (USFWS), the National Aeronautics and Space Administration (NASA) and the U.S. Geological Survey (USGS).

Variable	Data Description	Units	Definition	Spatial Resolution	Temporal Resolution	Data Source	# Predictors	Predictors Used to Develop Models
Mesophotic hard corals (MHC)	1,327 presences + 14,439 absences = 15,766 points	N/A	Presence/absence of hard corals (by genus) between 30 and 150 m in depth. The spatial uncertainty of the location is ± 15 to 100 m.	Mean nearest neighbor distance of points = 13 m; Mean height above seafloor unknown	09/09/2004–07/17/2010	NOAA, University of Hawai‘i, Bishop Museum, USFWS, State of Hawai‘i DAR	-	-
Seafloor Complexity	Aspect*	Degrees	Compass direction of maximum slope calculated using ArcGIS's Aspect tool.	10x10, 25x25* and 200x200* m	N/A	USGS, University of Hawai‘i	1 x 3 = 3	(3) Aspect at 10x10, 25x25, 200x200 m
	Depth*	Meters	Water depth of seafloor.	10x10, 25x25* and 200x200* m	N/A	USGS, University of Hawai‘i	1 x 3 = 3	(3) Depth at 10x10, 25x25, 200x200 m
	Bathymetric Position Index (BPI)*	Unitless— = depressions, 0 = flat, + = ridges	Measure of where a reference location is (vertically) compared to locations surrounding it. BPI was calculated using the Benthic Terrain Modeler [27].	10x10, 25x25* and 200x200* m	N/A	USGS, University of Hawai‘i	1 x 3 = 3	(3) BPI at 10x10, 25x25, 200x200 m
	Curvature (General)*	1/100 meters— = concave + = convex	Measure of convexity/concavity of the landscape calculated using ArcGIS's Curvature tool.	10x10, 25x25* and 200x200* m	N/A	USGS, University of Hawai‘i	1 x 3 = 3	(3) General Curvature at 10x10, 25x25, 200x200 m
	Curvature (Plan/Cross-sectional)*	1/100 meters— = concave + = convex	Curvature of the surface perpendicular to the direction of maximum slope calculated using ArcGIS's Curvature tool.	10x10, 25x25* and 200x200* m	N/A	USGS, University of Hawai‘i	1 x 3 = 3	(3) Plan Curvature at 10x10, 25x25, 200x200 m
	Curvature (Profile/Longitudinal)*	1/100 meters— = concave + = convex	Curvature of the surface parallel to the direction of maximum slope calculated using ArcGIS's Curvature tool.	10x10, 25x25* and 200x200* m	N/A	USGS, University of Hawai‘i	1 x 3 = 3	(3) Profile Curvature at 10x10, 25x25, 200x200 m
	Rugosity*	Unitless	Ratio of surface area to planar area calculated using DEM Surface Tools [28]	10x10, 25x25* and 200x200* m	N/A	USGS, University of Hawai‘i	1 x 3 = 3	(3) Rugosity at 10x10, 25x25, 200x200 m
	Slope*	Degrees	Maximum rate of slope change calculated using ArcGIS's Slope tool.	10x10, 25x25* and 200x200* m	N/A	USGS, University of Hawai‘i	1 x 3 = 3	(0) Excluded because it was highly correlated with Rugosity
	Slope of Slope*	Degrees of Degrees	Maximum rate of maximum slope change calculated using ArcGIS's Slope tool.	10x10, 25x25* and 200x200* m	N/A	USGS, University of Hawai‘i	1 x 3 = 3	(3) Slope of Slope at 10x10, 25x25, 200x200 m
Light Availability	Euphotic Depth Zone	Meters	Depth of the euphotic zone derived using the MODIS Aqua sensor and the Morel model. The euphotic zone is defined as the area where photosynthetically active radiation (PAR) levels are > 1%. PAR is the spectral range of sunlight (400–700 nm) that organisms can use during photosynthesis.	4x4 km (Krigged 10x10 m)	2004–2010 (Grand mean, minimum, maximum, standard deviation)	NASA	4	(2) Grand mean & standard deviation
Water Temperature	Sea Surface Temperature (SST)	Degrees Celsius	Temperature of the sea surface during the daytime as measured by MODIS Aqua sensor.	4x4 km (Krigged to 10x10 m)	2004–2010 (Grand mean, minimum, standard deviation)	NASA	3	(2) Grand mean & standard deviation
Currents	Modeled tidal current velocity at depth	Centimeters/Second	Tidal current velocities (based on seasonal mean water stratification) modeled hourly and averaged over one year.	1x1 km at 35 & 85 m depths (Resampled to10x10 m)	Annual mean, maximum, variation in speed	University of Hawai‘i [24,25]	3 x 2 = 6	(3) Mean (35 & 85 m), variation (35 m)
Geographic	Distance to Shoreline*	Meters	Distance to the shoreline calculated using ArcGIS Euclidean Distance tool.	10x10, 25x25* and 200x200* m	N/A	GIS Derived	1 x 3 = 3	(3) 10x10, 25x25, 200x200 m
						Total # Predictors	43	34

Using ArcGIS, each predictor was re-projected to the same coordinate system (i.e., NAD83 UTM 4 North), clipped to the same geographic extent, and saved to an ESRI ASCII (.asc) file for use in the MaxEnt software package [15, 26]. Eight metrics describing the complexity of the seafloor were calculated from a 10x10 m depth surface using several tools in ArcGIS [27, 28] (Table 1). These metrics were chosen based on previous studies that suggested they were potentially influential predictors of hard coral presence [29, 30, 31, 32]. The mean of these complexity metrics were also computed at two additional spatial scales (i.e., inside moving circular windows with radii of 25 and 200 m) in ArcGIS. Broad scale BPI (Bathymetric Position Index) was calculated with the inner radius of 10 m and outer radii of 25 m and 200 m [25]. Predictors at these different scales were included to explore the influence of spatial scale on MHC distributions, since organisms typically respond to their environment at multiple spatial scales [33, 34, 35].

Since the spatial resolution of the oceanographic variables (i.e., light availability, sea surface temperature, and tidal currents) did not match those of the complexity and geographic metrics, additional steps were required before they could be included in the modelling process. The tidal current surfaces were resampled from 1x1 km to 10x10 m using cubic convolution. Grand mean, minimum, maximum and standard deviation were calculated from 2004 to 2010 for the annual mean euphotic depth surfaces (i.e., PAR availability) and sea surface temperature (SST) surfaces. These 4x4 km grid surfaces were converted to points, and ordinary kriging was used to develop geostatistical predictions at 10x10 m. Table 2 describes the theoretical variograms used to develop these geostatistical surfaces. These parameters minimized the root mean square error of the final surfaces. Kriging was used (instead of resampling) to change the spatial resolutions of the SST and light availability predictors because several nearshore data gaps existed and needed to be filled around the islands of Maui, Lāna‘i, Moloka‘i, and Kaho‘olawe.

**Table 2:** Kriging parameters. The input parameters used to develop 10x10 m surfaces for euphotic depth and sea surface temperature using ordinary kriging. These parameters minimized the root mean square error of the final surfaces.

Variable	Semi-variogram Model	Nugget	Range	CV Root Mean Square Error
Euphotic Depth (Mean)	Gaussian	5.00E-35	15,306	1.03
Euphotic Depth (Stdev)	Stable	0	23,348	0.53
SST (Mean)	Gaussian	0.002	69,105	0.06
SST (Stdev)	Gaussian	0	7,984	0.04

**Fig 3:**
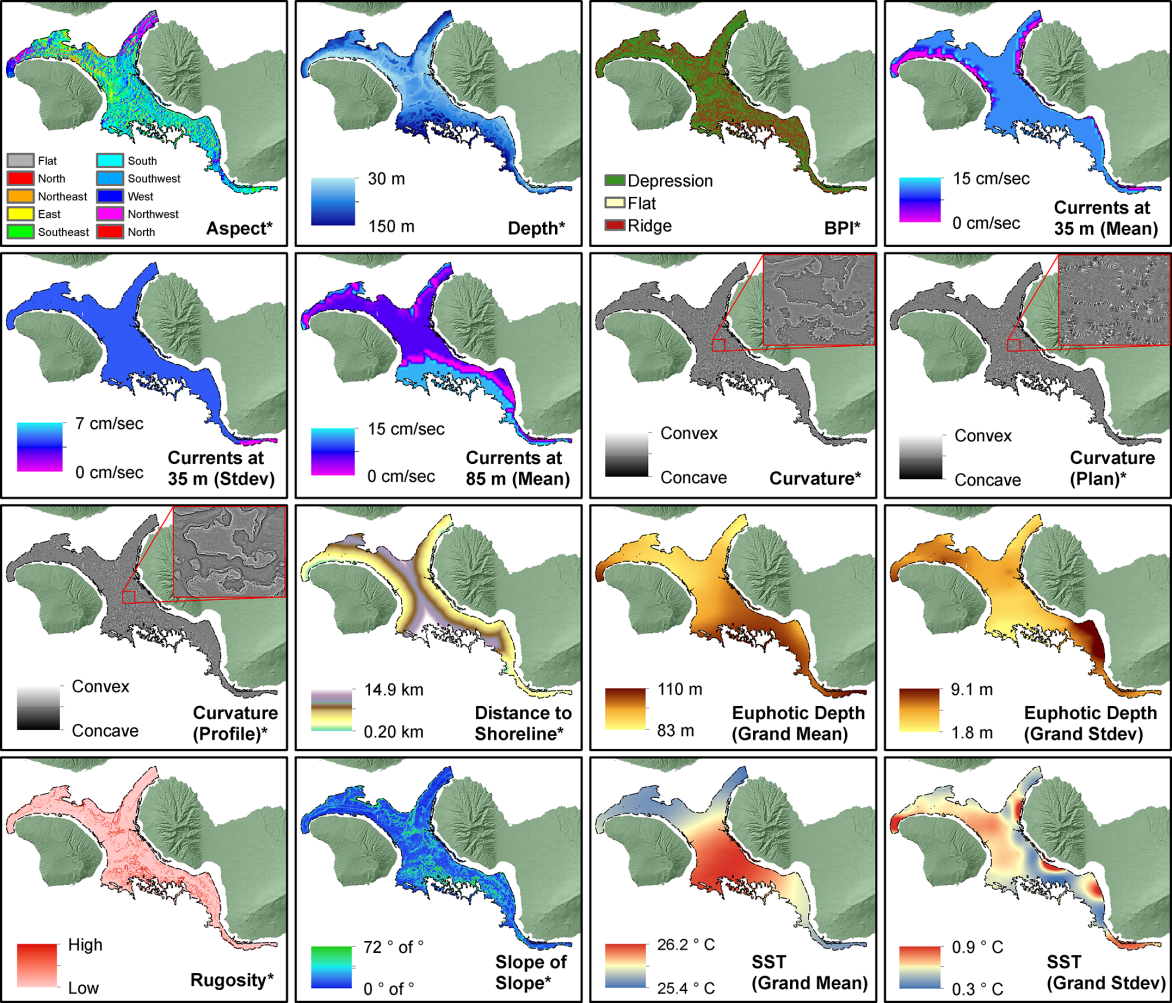
Maps of environmental predictors. These maps show the 10x10 m predictors that were used to develop MaxEnt models and spatial predictions for Montipora, Leptoseris and Porites. Asterisks denote predictors that were included at multiple spatial scales. The red inset boxes show fine-scale detail for predictors that are difficult to see.

A total of 43 predictors were considered for inclusion in the MaxEnt modeling process. The correlation of these 43 predictors was explored at 30 spatially independent locations using Spearman’s Rank tests. Even though MaxEnt is fairly robust in dealing with correlated predictors, nine predictors were removed from further analysis that were significantly (p≤0.05) and highly correlated (r2>0.85) with other predictors to reduce the amount of computational time needed to create spatial predictions. Predictors calculated at coarser spatial scales (i.e., 25 x 25 and 200 x 200 m) were an exception to this rule, and were left in the analysis to explore the influence of spatial scale on MHC distributions. The remaining 34 predictors were included in the modeling process (Fig 3).

### Predicting Mesophotic Hard Coral Distributions

#### MaxEnt

Maximum Entropy modeling software (MaxEnt 3.3.3e) was used to create spatially explicit predictions for *Montipora*, *Leptoseris* and *Porites* occurrence. MaxEnt [15, 24] is a modeling technique that predicts the distribution of organisms using presence-only data. It makes these predictions by analyzing the distribution of environmental variables associated with the organisms’ presence to find other areas that meet all of these environmental constraints (without making any assumptions about what is not known).

Even though absence data was available for this study area, MaxEnt was chosen over other modeling approaches that use both presence and absence data for two main reasons. First, this project and modeling approach was designed to be expanded to areas around the entire MHI. This future expansion will require mesophotic coral data to be compiled from various platforms of opportunity. It is likely that the majority of this opportunistic data will be presence-only (or diverse datasets that will need to be standardized to presence-only), making MaxEnt a more broadly applicable approach and a more likely candidate for future MHC modeling efforts in the MHI.

In addition, MaxEnt compares favorably to presence and absence modeling techniques including generalized linear models (GLM), generalized additive models (GAM), boosted regression trees (BRT), genetic algorithm for rule-set prediction (GARP), multivariate adaptive regression splines (MARS) and environmental niche factor analysis (ENFA) [15, 23, 36, 37, 38, 39]. Specifically, Elith et al. (2006) [36] found that MaxEnt was in the highest preforming group [in terms of Area Under the Curve (AUC), correlation and Cohen’s kappa] when predicting the distribution of terrestrial species in several locations around the world. These results suggest that MaxEnt is capable of producing spatial predictions that are (at the very least) as accurate as some of the more complicated, data and labor intensive modeling techniques available.

#### Model development

Ten MaxEnt models (i.e., model ensembles) were developed using randomly selected subsets of data for each of the MHC genera (i.e., *Montipora*, *Leptoseris* and *Porites*). Multiple models were created for each genus to avoid fitting one model too closely to the data, and to better understand and quantify the stability of, and uncertainty associated with, MaxEnt’s variable selection and predictive performance. The same 34 predictors and input parameters were used for all the models. The majority of MaxEnt’s input parameters were left at their default value, since few guidelines exist for optimizing these parameters [15, 24], and even fewer exist for optimizing these parameters for modeling MHCs [23, 40]. The few model parameters that were changed include: Random seed (on), Replicated run = Subsample, Random test percentage (i.e., out-of-bag test points) = 50%, Replicates = 10, Maximum iterations = 1,000, Regularization = 1 and Default prevalence = 0.038, 0.039 and 0.007 (for *Montipora*, *Leptoseris* and *Porites*, respectively). The prevalence of each genus was derived from the *in situ* presence/absence data. The “random seed” refers to the randomly chosen starting point used to subset the data for model training and cross validation. A randomly chosen starting point and random 50% of the data were used to reduce the likelihood of a single starting point (or set of points) biasing the overall model results. The regularization value was determined heuristically, and values = 0.0001, 0.01, 0.1, 1, 2.5 and 5 were tested. A regularization value of 1 was chosen because it produced models with the highest test AUCs. Response curves, jackknife analysis, and spatial predictions were developed for each model replicate. These results were averaged by genus to produce the final MaxEnt performance metrics and spatial predictions for *Montipora*, *Leptoseris* and *Porites*.

The spatial predictions output by MaxEnt denote the average probability of occurrence values for each genus (since the prevalence of each genus was estimated within the study area). Probability of occurrence denotes the statistical likelihood (between 0% and 100%) that a genus is present at a given location. It is important to note that probability of occurrence is different from relative habitat suitability, which is the standard output for MaxEnt when prevalence of an organism is unknown. Habitat suitability describes (on a relative 0–1 scale) whether environmental conditions in one location are similar to those at other locations where an organism was observed. Here, the availability of presence and absence data provided an opportunity to calculate empirical prevalence for MHCs, and evaluate biases associated with MaxEnt predictions.

#### Model performance

We assessed the discrimination capacity (i.e., its ability to distinguish between classes) and reliability (i.e., the agreement between predicted and observed values) of the models [41, 42]. Reliability of the spatial predictions was evaluated using mean absolute error (MAE). MAE measures the average magnitude of the predictive errors (independent of their direction). It was calculated by intersecting and subtracting each genus’ probability of occurrence prediction replicate (with values ranging from 0 to 1) from spatially independent *in situ* presence (= 1) and absence (= 0) data. The presences used to calculate MAE were the out-of-bag (50%) test points set aside by MaxEnt before each model run. The absences used to calculate MAE were set aside at the beginning of the modeling process. Spatially autocorrelated *in situ* points were identified using variograms (model = spherical, nugget = 0.5, partial sill = 0.04, and range = 107 meters), and points closer than 107 m were removed using Matlab. The absolute values of these residual model errors (from the 10 model replicates) were averaged to calculate the overall MAE for each final spatial prediction.

Receiver Operative Characteristic (ROC) curves were used to evaluate the discrimination capacity of the models. ROC curves measure a model’s performance by comparing its sensitivity (i.e., true positive prediction rate) to its specificity (i.e., false positive prediction rate) over the continuous range of predicted values. The area under each ROC curve (known as the Area Under the Curve or AUC) was also calculated. AUC values ranging from 0.5 to 0.6 suggest the model is no better at discriminating classes than random chance; values from 0.6 to 0.7 denote “poor” model performance; 0.7 to 0.8 denote “acceptable” model performance; 0.8 to 0.9 denote “excellent” model performance, and values greater than 0.9 denote “outstanding” model performance [43]. ROC curves and AUCs were generated by the MaxEnt software package for each model replicate using the out-of-bag test points. Separate ROC curves and AUCs were also generated for each model replicate from the presence and absence data (used above to calculate MAE) in R software. AUC was calculated in two different ways because MaxEnt uses background points rather than true absences to estimate specificity, which makes comparing models for commonly found genera to relatively more rare genera difficult [15]. Background points are random samples of the full spectrum of environmental conditions in the study area without regard to the presence or absence of an organism. The use of background points (and not true absences) means that the maximum possible test AUC value depends on what fraction of the study area is occupied by the species (maximum possible AUC = 1—(a/2), where a is the fraction of grid cells occupied by the species) [44].

#### Spatial distribution of model uncertainty and errors

ROC curves and AUC values measure model performance but they do not describe the spatial distribution of model uncertainty and errors [45, 46, 47]. Analyzing the spatial location and clustering of uncertainty and errors can be important because it may offer clues about sampling bias or missing environmental covariates that are influencing the distribution of an organism [1, 35, 47, 48, 49]. Here, predicted uncertainty was quantified for each genus by calculating the standard error among the MaxEnt 10 model replicates. The magnitude and direction of modeling errors were quantified from the same data used to calculate MAE. However, instead of averaging these residual errors across the entire prediction (as was done for MAE), spatially coincident points were averaged, and their spatial distribution and clustering were evaluated using cluster and outlier analysis in ArcGIS’s Spatial Analyst Toolbox. This analysis identified statistically significant spatial clusters of high values, low values, and outliers using inverse distance weighting squared and the Anselin Local Moran's I statistic.

#### Contribution of predictor variables

Two metrics (i.e., jackknife AUC analysis and single variable response curves) were used to quantify the contribution of each environmental variable to each model and its performance. These two metrics were evaluated together to determine which variable(s) were the most influential predictors and at what thresholds. Response curves were used to describe how probability of occurrence values changed in the context of a hypothetical single-variable model. Jackknife analysis measures the contribution of each variable to a model’s gain (goodness of fit), and its impact on test AUC values using only one variable at a time, and excluding one variable at a time. This process of inclusion and exclusion isolates the contribution of each predictor variable from the other variables, and describes whether a particular variable improves or degrades the performance of a model. A large drop in AUC indicates that the model is heavily dependent on that particular variable, while a small drop indicates that the predictor does not contribute much new information (i.e. the information it contains is redundant) [50]. The predictors with the top six single variable jackknife test AUC values are discussed here.

## Results

### Spatial Predictions of Occurrence

**Fig 4:**
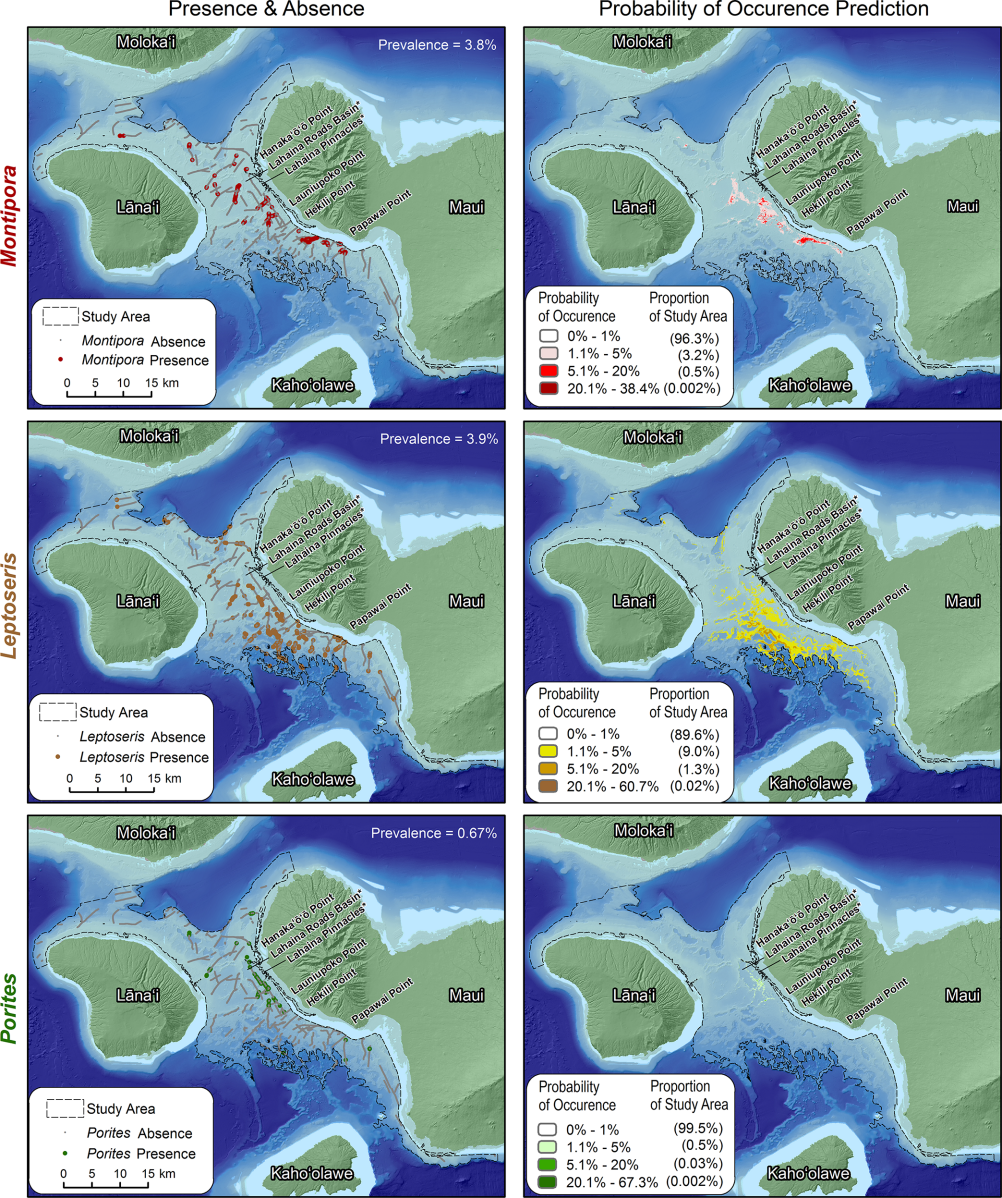
Observed and predicted MHC distributions. The maps on the left show the location of *Montipora*, *Leptoseris* and *Porites* corals, and the maps on the right show the predicted distributions of *Montipora*, *Leptoseris* and *Porites* habitats. These predicted distributions were created by spatially averaging the 10 model replicates for each genus.

*Montipora*, *Leptoseris* and *Porites* were observed in 3.8% (i.e., 605/15,766), 3.9% (i.e., 617/15,766) and 0.67% (i.e., 105/15,766) of the field observations, respectively. The average test AUCs calculated by MaxEnt and separately in R indicated ‘excellent to outstanding’ overall performance for the *Montipora*, *Leptoseris* and *Porites* models (Table 3). *Montipora* and *Leptoseris* were predicted to be rare throughout the study area with spatial predictions denoting a 0–1% chance of it occurring in 96.3% and 89.6% of the study area. *Porites* was predicted to be even less common and less likely to occur than *Montipora* and *Leptoseris*, with spatial predictions denoting a 0–1% chance of it occurring in 99.5% of the study area. For *Montipora*, probabilities for the remaining 3.7% of the study area ranged from 1.1% to 38.4%, with highest probabilities concentrated in four areas in the middle of the ‘Au‘au Channel. These areas include near the Lahaina Roads Basin, ~4.2 km off Launiupoko Point, and ~3.1 km off Hekili Point and ~3.7 km off Papawai Point (Fig 4). For *Leptoseris*, probabilities for the remaining 10.4% were higher, ranging from 1.1% to 60.7%, and were concentrated in the south/central region of the ‘Au‘au Channel off of Hekili and Papawai Points (Fig 4). High probabilities were also found along many edges of drowned basins and ridge tops with a hotspot located at 20°46’ N, 156°41’ S. For *Porites*, probabilities for the remaining 0.5% of the study area ranged from 1.1% to 67.3% and were focused region along the eastern side of the ‘Au‘au Channel. These regions included areas between Lahaina Roads Basin and Hekili Point with the main hotspot south of the Lahaina Pinnacles (Fig 4).

**Table 3:** Test AUC values for *Montipora*, *Leptoseris* and *Porites*. Test AUC values computed by MaxEnt using the 50% out-of-bag presences and background points, and a separate AUC computation in R using true absences and the same 50% out-of-bag presences used by MaxEnt.

	*Montipora* Test AUC		*Leptoseris* Test AUC		*Porites* Test AUC	
Data Used	Average	Std. Error	Average	Std. Error	Average	Std. Error
Presences & Background Points	0.97 (adjusted maximum = 1-[0.04/2] = 0.98)	0.002	0.93 (adjusted maximum = 1-[0.10/2] = 0.95	0.002	0.95 (adjusted maximum = 1-[0.005/2] = 0.99)	0.004
Presences & Absences	0.95	0.002	0.89	0.003	0.93	0.006

### Spatial Prediction Uncertainty & Error

**Fig 5:**
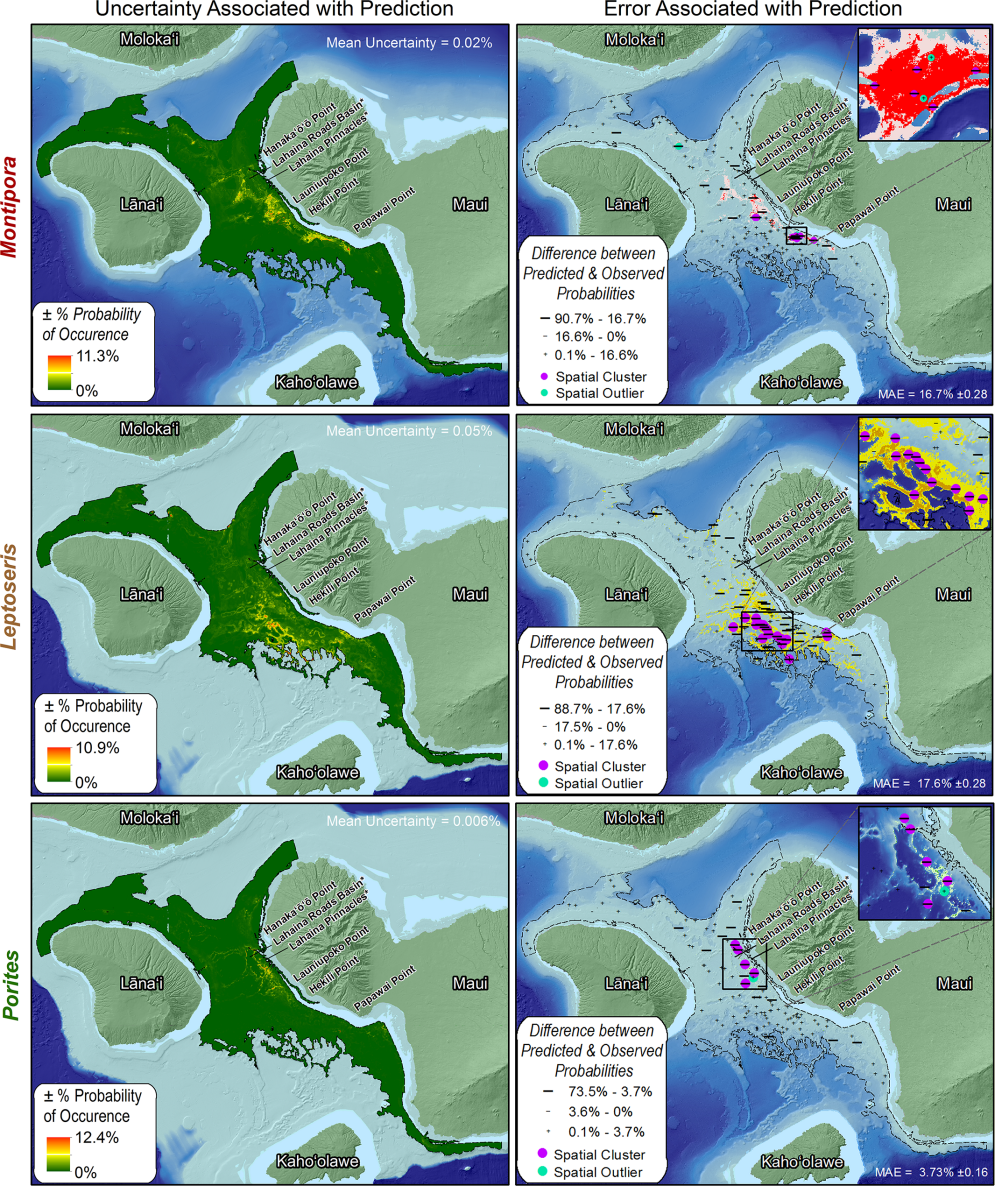
Uncertainty and error associated with MHC predictions. These maps show the spatial uncertainty (i.e., standard error) and spatial errors (i.e., difference between observed and predicted values) associated with *Montipora*, *Leptoseris* and *Porites* spatial predictions averaged across the 10 model replicates. Errors were divided into classes based on the MAE, and summarized by ROV transect for display purposes. Each +/- symbol on the map denotes the mean error along a single ROV transect.

The average uncertainty (i.e., standard error) for the *Montipora*, *Leptoseris* and *Porites* predictions were 0.02%, 0.05% and 0.006%, respectively. The highest uncertainties for each genus were roughly co-located with its highest probability of occurrence values (Fig 5). The MAE for the *Montipora*, *Leptoseris* and *Porites* predictions were 16.7% ±0.28, 17.6% ±0.28 and 3.73% ±0.16, respectively. For each genus, there was relative agreement among the majority (i.e., 70.8%, 69.3% and 92.5%) of observed and predicted probabilities of occurrence values (i.e., difference between them was < MAE). For the remaining records, 0.0%, 0.1% and 0.3% were positive and ≥MAE, and 29.4%, 30.6% and 7.2% were negative and ≥MAE for *Montipora*, *Leptoseris* and *Porites*, respectively. These numbers indicate that MaxEnt more frequently under-predicted (versus over-predicted) the probability of occurrence for all three genera (Fig 5).

For *Montipora*, it is interesting to note that most of the errors ≥MAE were located in a single area of high probability of occurrence, south of Hekili Point. The cluster analysis highlighted this spatial pattern by identifying six significant negative error clusters and two significant positive error outliers. One large (≥MAE), negative outlier was also northeast of Lāna‘i. For *Leptoseris*, the cluster analysis identified approximately 15 significant negative error clusters south west of Hekili Point. These significant clusters were located between 75 and 110 m depths on the edge of steep slopes, which dropped off into basins to the southwest. Large (≥MAE) negative error clusters were also located offshore of Papawai Point. For *Porites*, the cluster analysis identified five significant, large (≥MAE) negative error clusters between Hanaka‘ō‘ō and Launiupoko Points. Four of these clusters were located in areas of low (<1%) probability of occurrence. The remaining cluster was located in an area with higher (1.1%-5%) probabilities of occurrence adjacent to one small (≤MAE) positive error cluster south of Lahaina Pinnacles.

### Defining Suitable Habitats

**Fig 6:**
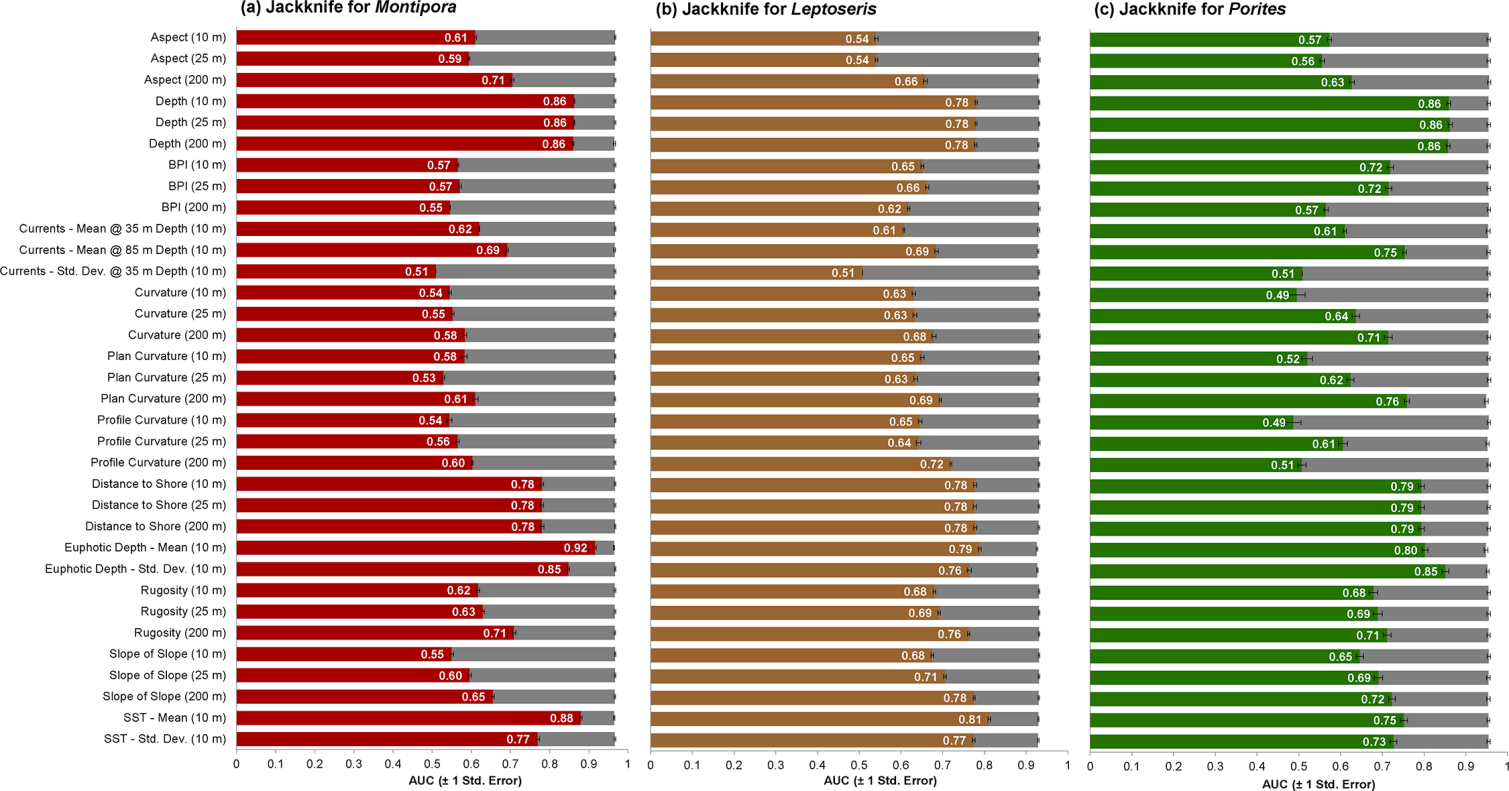
Jackknife analysis. Jacknife analysis for *Montipora*, *Leptoseris* and *Porites* showing the mean AUC when a single variable is used to develop a model (red, brown and green bars) or excluded from the modeling process (gray bars). This process of inclusion and exclusion isolates the contribution of each predictor variable from the other variables, and describes whether a particular variable improves or degrades the performance of a model. The AUC value for a single variable model is depicted inside the bars. The error bars denote one standard error (based on 10 model iterations).

**Fig 7:**
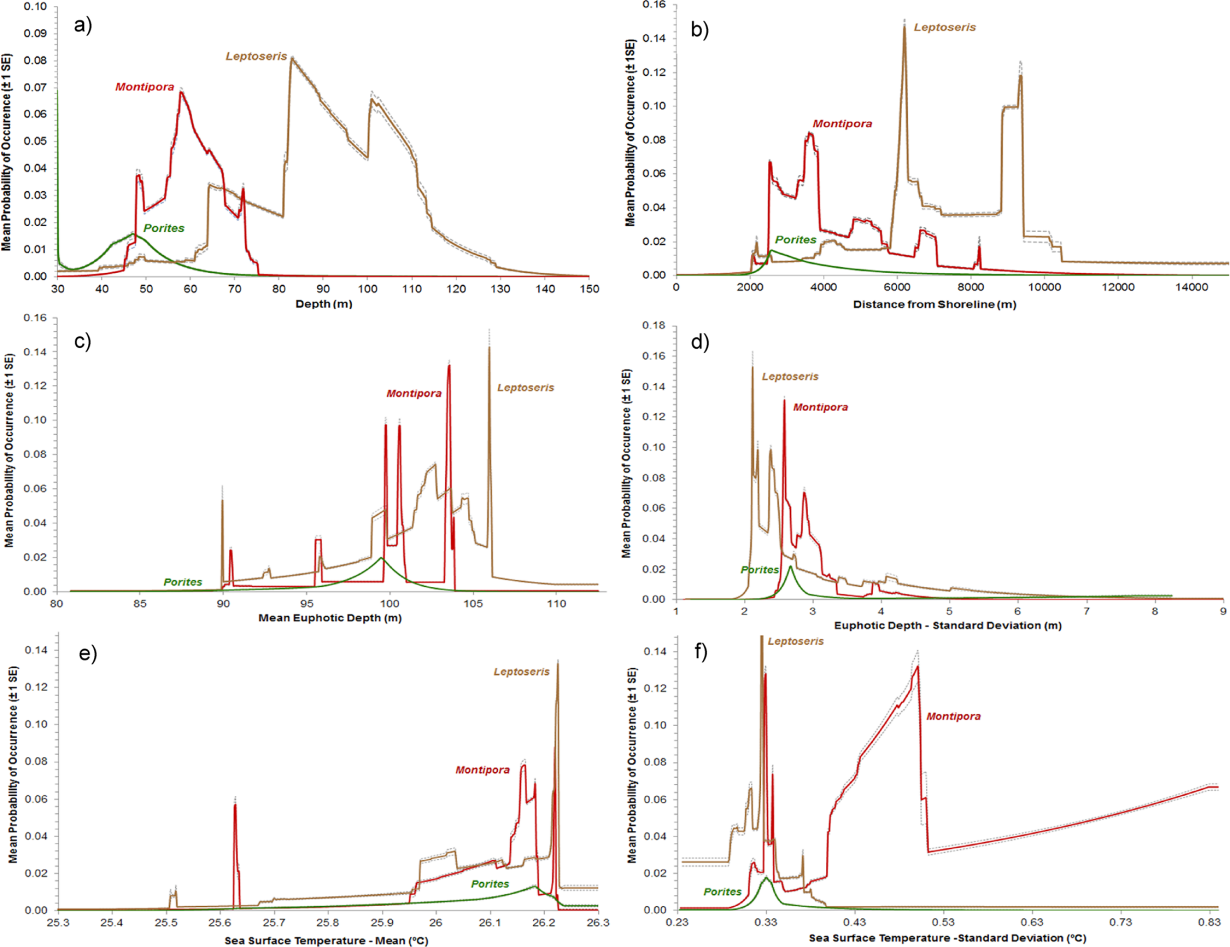
Single variable response curves. The single response curves for the six most important environmental variables for predicting the occurrence of *Montipora*, *Leptoseris* and *Porites*: a) depth (meters), b) distance from shoreline (meters), c) mean euphotic depth (meters), d) standard deviation of euphotic depth (meters), e) mean sea surface temperature (°C), and f) standard deviation of sea surface temperature (°C).

*Montipora*, *Leptoseris* and *Porites* shared several variables that were influential in their models (as identified through the jackknife tests) (Fig 6). Shared, important variables included depth, distance from shore, euphotic depth (mean and standard deviation) and SST (mean and standard deviation). Slope of slope and rugosity (at 200 m) were also important for predicting Leptoseris. That being said, the Montipora, Leptoseris or *Porites* models were not dependent upon any single variable (or spatial scale), since their test AUCs did not decline dramatically when single variables were iteratively excluded from the modelling process. Comparisons across the genera offer some insight into how they partition the space differently in the study region. Notably, peak probability of occurrence values were shallowest for *Porites* (~47 m), deeper for *Montipora* (~60 m), and covered a broad, but deeper range of depths for *Leptoseris* (~85 m) (Fig 7A). Peak probability of occurrence values for distance to shore were shortest for *Porites* (~2.4 km), slightly farther offshore for *Montipora* (~4 km), and furthest offshore for *Leptoseris* (peaks at ~6 and ~9 km) (Fig 7B). It is important to note that no records were collected closer than 2.4 km from shore. In contrast, to depth and distance to shore, mean and variance of euphotic depth and mean SST showed broadly overlapping occurrences for all 3 genera. *Leptoseris* had the deepest peak at euphotic depth of 106 m, followed by *Montipora* at 103 m and *Porites* at 99 m (Fig 7C). *Leptoseris* showed peak probabilities in areas with lower variability in euphotic depth than *Porites* and *Montipora* (Fig 7D). *Montipora* showed peak probabilities in areas with lower SST and higher variation of SST than *Porites* or *Leptoseris* (Fig 7E and 7F).

Comparing model results can be misleading when based on organisms with markedly differing prevalence. Fortunately, all the models created here were for comparatively rare biota (i.e., occurred in <4% of the records). Qualitative evaluation revealed that the three genera were divided in space, and occupied somewhat different parts of the study area. Probabilities for all three genera were only >0% in a small area south of the Lahaina Pinnacles. Outside the area of overlap, *Porites* was predicted to be dominant 2–3 km off of Lahaina, *Montipora* 3–4 km south of Hekili Point, and *Leptoseris* along the ridges 6–9 km offshore around Hekili Point. Many areas are evident where the three genera occupy adjacent, but not overlapping bands of suitable habitat. This highlights habitat partitioning among genera along environmental gradients on the ridges and basin walls in the study area.

## Discussion & Conclusions

MaxEnt produced reliable spatial predictions for *Montipora*, *Leptoseris*, and *Porites* as measured by the high average test AUC values. The three spatial predictions developed show that highly probable locations for MHCs are both relatively rare and distributed unevenly in the study area. No single environmental variable tested here fully explained why suitable MHC habitat was clustered in certain locations. However, six predictors were identified as being important for predicting probable habitat across all of the MaxEnt models. Probability of occurrence values for each coral model overlapped across the range of predictor values examined here, although peak probabilities occurred at different values of the predictors for different groups. The more detailed discussion (below) about these predictors, the differences and similarities in peak probabilities among genera, and their influence on habitat predictions by geographic region offers insight into the factors governing distribution of MCEs.

### Montipora

The majority of probable habitat for *Montipora* is predicted to be on the southeastern side of the ‘Au‘au Channel between Lahaina Roads Basin and Papawai Point. This southeastern area is characterized by relatively warmer (at the surface), moderately deep, and less turbid waters than parts of the north, west, and southwest ‘Au‘au Channel and the study area as a whole. Euphotic depth, which is a proxy for both PAR and turbidity, also appears to be less variable in this southeastern location than other parts of the study area, remaining consistent (> 1% PAR depth within ± 2.3 m) over a six year period from 2004 to 2010. Water temperature measurements taken near this area in 2001 indicate that this layer of warm water (around 26°C) may be fairly stable down to about 60 m, after which it drops to around 23°C at 111 m [51]. The variation in temperature profiles down to 60 m is similar to the temperature variation seen in the SST imagery (i.e., ± 0.9°C). These numbers and patterns suggest that SST may be a proxy for warmer water down to approximately 60 m in depth, although more measurements are needed to better characterize the influence of internal waves, tides, and seasons on the spatial and temporal heterogeneity of water temperature at depth [52, 53]. These environmental trends suggest that on the whole *Montipora* prefers relatively warmer, moderately deep waters that remain optically clear and stable through time. These habitat preferences are highlighted by the jackknife results, which show the highest probability of occurrence values at moderate depths (~59 m) and in warmer (26.15 to 26.22° C at the surface), clearer (>1% PAR depth of 103 m) waters.

These depth preferences and thresholds are in close agreement with the findings of Rooney et al. (2010) [8], which reported that *Montipora* was one of the most common coral genera found in 50 to 80 m of water. However, these thresholds differ from the results reported by Kahng and Kelley (2007) [10], which found *Montipora* to be rare in the 50 to 80 m range. It is interesting to note that the ROV transects in the ‘Au‘au Channel analyzed by Kahng and Kelley did not intersect with the spatial distribution of highly probable *Montipora* habitat predicted by MaxEnt. This geographic mismatch suggests that Kahng and Kelley may have sampled in areas with relatively poor ambient conditions for *Montipora* or a different range of environmental variables than were evaluated in this study. It is very likely that the environmental predictor variables (especially depth, distance to shore and SST) are proxies for other environmental conditions favorable to *Montipora* recruitment and growth. For example, depth and distance to shore are most likely correlated proxies for light availability, since generally speaking, the seafloor becomes deeper and the water becomes less turbid further from land. Kleypas et al. 1999 [54] found this same positive correlation between PAR and distance from shore when comparing nearly 1,000 reef locations from around the world. High SST may also be a proxy for calm, lower turbidity waters. The area of highest SST overlaps with the relatively windless, lower rainfall, lower wave energy, leeward side of west Maui.

### Leptoseris

The majority of probable habitat for *Leptoseris* is predicted to be in the southern part of the ‘Au‘au Channel close to where it meets with the Kealaikahiki Channel. This area has similarly warm water temperatures as in the eastern and southeastern areas discussed above. However unlike these two areas, the southern part of the ‘Au‘au Channel had deeper waters on average. It also has the most consistently warm (26.2°C) and clear waters (> 1% PAR depth within ± 2 m) compared to any other part of the ‘Au‘au Channel or study area as a whole. Also, the water temperature at depth most likely remains within the tolerated range for *Leptoseris* (i.e., > 19°C) down to 120 m in this area [10, 51, 54]. Collectively, these environmental trends suggest that *Leptoseris* prefers slightly deeper, substantially less turbid and less variable waters (in terms of turbidity and, possibly, temperature) than *Montipora*. These habitat preferences are quantified by the jackknife results and response curves, which show the highest probability of occurrence values for *Leptoseris* occurred at the deepest depths (84 m), in the least turbid (>1% PAR depth at 106 m) and least variable waters (>1% PAR within ± 2.1m and SST ±0.33°C) in comparison to the other genera models.

These depth preferences and thresholds are in close agreement with the findings of Kahng et al. 2010 [9], which reported that *Leptoseris* corals were commonly found in the deepest parts of the mesophotic zone across the Pacific. They also agree with those of Rooney et al. 2010 [8] and Kahng and Kelley (2007) [10], which documented that the hard substrata between 80 to 90 m was dominated by aggregations of *Leptoseris*. However, *Leptoseris* has been recorded at deeper depths in the MHI, including at 131 m and 153 m near Penguin Banks and Kealakekua Bay, respectively [10, 55]. *Leptoseris*’s presence at these exceptionally deep depths suggest that temperature is not a limiting factor for its growth in Hawai‘i [10, 54], even though temperature at the water surface was identified as an important predictor in this modeling process. Therefore, SST (especially standard deviation) is most likely a proxy for another environmental variable describing the stability of the water conditions in the area. The availability of PAR was identified as one of the least variable conditions by MaxEnt. *Leptoseris*’ preference for less turbid and more optically stable waters also aligns with the findings of Kühlmann 1983 [56], which showed that corals with flat morphologies (like *Leptoseris*) are particularly sensitive to sedimentation [9]. Flat morphologies are also less effective for passive suspension feeding than the branching structure of many azooxanthellate corals [9]. Despite potentially being less effective at heterotrophy and more susceptible to sedimentation, the flat, plate-like morphologies and dark brown pigmentation of *Leptoseris* and other mesophotic corals have advantages, including being specialized for capturing the maximum amount of light [9, 10].

In addition to being found most commonly at the deepest depths, *Leptoseris*’ highest probability of occurrence values were located the furthest from shore (6.1 km), likely because distance to shore is correlated with and a relatively good proxy for increasing depth, decreasing turbidity and reduced variability in PAR [54]. In addition to these six predictors, rugosity and slope of slope (both at 200 m scales) were also important for predicting *Leptoseris* distributions. None of the other MaxEnt models identified seafloor complexity as being important. The inclusion of these morphometrics suggests that some other variable associated with high complexity (specifically available hardbottom) may also play a role in determining the distribution of *Leptoseris*. This pattern is in keeping with Rooney et al. 2010 [8], which noted that *Leptoseris* and *Montipora* both inhabited mainly hardbottom habitat (although they were also present in some softbottom areas).

### Porites

The majority of probable habitat for *Porites* is predicted to be on the eastern side of the ‘Au‘au Channel between Hanaka‘ō‘ō and Launiupoko Point. Similar to the southeastern area described above, this area is characterized by relatively warmer, slightly shallower and less turbid waters than found in other parts of the study area. Based on the temperature profiles reported above [51], temperature most likely remains within the tolerated range for some species of *Porites* down to over 100 m in this area [10, 54]. It is also important to note that turbidity levels vary slightly more (> 1% PAR depth within ± 2.7 m) and SST varied slightly less (±0.33°C) in this area than in locations further to the south where the ‘Au‘au and Kealaikahiki Channels meet. Collectively, these environmental trends suggest that *Porites* prefers shallower waters than *Montipora* and *Leptoseris*. These depth preferences agree with the findings of Rooney et al. 2010 [8], which reported that depths from 30 to 50 m were dominated by several shallow water coral species, including *Porites lobata*. They also agree with the results of Grigg 2006 [51], which reported that while *Porites lobata* can grow at depths up to 100 m, it is more common to find this species at depths shallower than 50 m.

Given these consistent depth preferences, it is likely that *Porites* is limited by the availability of PAR more so than by temperature [9, 51] or by any other predictor included in this study. This relationship is not new, as the depth limit of reef building corals has long been associated with decreasing PAR [54, 57, 58]. Even though *Porites* distributions may primarily be light-limited, the single variable response curve for standard deviation of euphotic depth (Fig 7D) also suggests that *Porites* can tolerate slightly more turbid (although still exceptionally clear) waters than either *Montipora* or *Leptoseris*. This increased resiliency agrees with the findings of Piniak 2007 [59], who reported that *Porites lobata* experienced less tissue damage from sedimentation than did *Montipora capitata* because of its more rugose morphology. These habitat preferences are quantified by the response curves, which show the highest probability values for *Porites* occurred at comparatively shallow depths (43 m; Fig 7A) and in the most turbid (>1% PAR depth at 99 m; Fig 7C) and most variable waters (>1% PAR varied by 2.6 m; Fig 7D) compared to any of the other models. *Porites*’ highest probability values were also located the closest to shore (2.4 km), likely because distances closer to shore are correlated with and a relatively good proxy for decreasing depth, increasing turbidity and increasing variability in PAR.

### *Montipora*, *Leptoseris* and *Porites*: Model Uncertainty and Error

Based on the above model outputs, it is clear that the distributions of *Montipora*, *Leptoseris* and *Porites* were not fully explained by the modeling process, given the high uncertainties and large (≥MAE) spatially clustered errors and outliers in some locations. The areas south of Hekili Point, southwest of Hekili Point and between Hanaka‘ō‘ō and Launiupoko Points are notable places where high probabilities of occurrence, high uncertainty, and large (≥MAE), spatially clustered errors and outliers co-occurred for the *Montipora*, *Leptoseris* and *Porites* predictions, respectively. The high values for these model metrics (i.e., probability of occurrence, uncertainty and error) most likely co-occurred because MaxEnt identified overlapping, but slightly different environmental envelopes for each model replicate. Different, overlapping envelopes were defined because different, randomly chosen training datasets were used to develop each model replicate used in the model ensemble. These envelopes may also have varied because of the positional uncertainty (± 15–100 m) associated with the training points. Such uncertainty means that, in some cases, the location of the training points were off by a maximum of 10 raster pixels (based on 10x10 m raster predictors). These two sources of variation and their impact on MaxEnt’s environmental envelope underscores the utility of a modelling ensemble approach, since this type of spatially-explicit uncertainty can help scientists and resource managers put confidence limits on their research and regulatory decisions and planning processes.

In addition differences among training datasets and model replicates, the high values for these model metrics also most likely co-occurred because *Montipora*, *Leptoseris* and *Porites* are responding to or impacted by ecological factors (e.g., disease, competition or recruitment), anthropogenic impacts (e.g., impacts from land based sources of pollution) or other environmental conditions (e.g., availability of uncolonized substrate) at finer temporal or spatial scales. It is difficult to identify which of these conditions (or combinations of conditions) best explain these anomalous areas. However, for *Montipora*, further analysis of the SST standard deviation surface showed that SST varied more widely south of Hekili Point (i.e., by about 0.5°C) than in any other location in the study area with high probabilities of occurrence for *Montipora*. This higher variation is also reflected in the single variable response curve for *Montipora*, which shows a spike in probability of occurrence that is approximately 0.2°C higher than *Leptoseris* or *Porites* (Fig 7F). While these temperature fluctuations are comparatively small [22], they may stress *Montipora* corals in this location. Thermal stress has been shown to impair coral growth [60], and to make corals more susceptible to other impacts from disease, human uses, and climate change [61, 62, 63, 64].

For *Leptoseris*, further analysis of the ROV data used for this modeling exercise showed that the area southwest of Hekili Point had higher (>50%) amounts of hardbottom than in any other location in the study area with high probabilities of occurrence for *Leptoseris*. Of these hardbottom areas, many were classified as rubble in the ROV data, particularly those closer to shore. These nearshore, hardbottom areas were not visible in the depth surface and seafloor complexity derivatives because rubble does not have a lot of relief (at the 10x10 m scale). However, rubble still provides important habitat for coral recruitment, and may help explain why MaxEnt under-predicted *Leptoseris* in this location. Including acoustic backscatter and/or an accurate map denoting hardbottom and softbottom locations may help capture additional, low-relief hardbottom areas, and help improve future *Leptoseris* predictions.

Lastly, for *Porites*, further analysis of the mean current surfaces showed that these errors roughly follow a boundary (vertically and horizontally) where water velocities change. This trend is also indirectly captured by the jackknife results for *Porites*, which identified the mean current predictor (at 85 m depth) as having a relatively high AUC (AUC = 0.75) compared to *Montipora* or *Leptoseris* (AUC = 0.69). Given that water velocities may be changing, this boundary may also denote the division between environments that are exposed to different amounts of wave energy and near bed shear stress. Wave exposure in particular has been identified as a major factor influencing the distribution and composition of coral reefs in Hawai‘i [65, 66, 67, 68, 69], and *Porites* species have been shown to be more abundant in environments with low-to-intermediate wave energies [70]. Including predictors denoting wave energy, bottom water velocity and/or near bed shear stress could help improve the spatial prediction for *Porites* in the future [70].

### Study Area Overall

Several physical conditions help make our study area (specifically the southeastern portion) an ideal place for MHCs [19, 66]. These conditions include having consistently good water clarity and being protected from strong wind and wave energy. Good water clarity is important because it affects the amount of PAR available at depth, while protection from strong wind and wave energy creates conditions favorable to faster rates of coral accretion [65, 71, 72]. Combined, these oceanographic and weather conditions create patches of comparatively warm, calm, clear waters that remain relatively consistent through time.

Although there were some slight differences among the MaxEnt models, these three environmental conditions (i.e., warm, clear and consistent water conditions) along with depth were the most important variables for predicting the distribution of *Montipora*, *Leptoseris*, and *Porites*. This trend may help explain why the distributions of *Montipora*, *Leptoseris*, and *Porites* fell primarily between Hanaka‘ō‘ō and Papawai Points, which appear to have the least variable local weather and water conditions in the study area. This environmental stability occurs for a number of reasons, including being wholly sheltered by the western Maui Mountains from the damaging North Pacific waves and strong trade winds. Shielding from strong winds and large waves may explain why a consistently warm mass of water sits between these two points almost year round. Water quality conditions appear to be equally stable in this location. This is probably due to a number of factors, including relatively lower amounts of rainfall and relatively lower amounts of urban and agricultural development in the adjacent coastal watersheds.

While these regional environmental conditions seem to explain mesophotic coral distributions very well at the scales examined in this study, it is also highly likely that several other biological and ecological factors, including predation, inter/intra-species competition and recruitment, have and are playing a significant role in shaping distributions, especially at finer spatial and temporal scales [1, 33, 73]. For example, Spalding 2012 [74] suggests that abundant macroalgae (e.g., *Caulerpa filicoides*, *Distromium* species) may compete with *Montipora* for space, and may have a measurable impact on *Montipora*’s spatial distribution in the ‘Au‘au Channel. Kahng and Grigg 2005 [75] reported that the introduced species, *Carijoa riisei*, competes with native black corals in the ‘Au‘au Channel. It is possible *Carijoa riisei* also competes with and impacts the distribution of other benthic organisms nearby. That being said, the influence of these biological and ecological forces on the spatial heterogeneity and species diversity of mesophotic corals is still poorly understood, and may change based on species, depth, location and scale. More research, especially *in situ* ecological manipulations, are needed to examine how these forces shape mesophotic coral community assemblages. This growing body of information can then be used iteratively to improve model specification in the future.

## Conclusions

Although significant research questions and data gaps remain, the MaxEnt predictive models created here performed well and can help researchers and managers explore questions related to spatial ecology and place-based conservation in the absence of timely, *in situ* information about MHCs. These models quantitatively showed that mesophotic coral distributions are concentrated between Hanaka‘ō‘ō and Papawai Points in the ‘Au‘au Channel because this area has some of the least variable environmental conditions in the study region, hosting warmer, clearer, and calmer water conditions almost year round. Although mesophotic corals are also responding to other environmental and ecological cues beyond the ones discussed here, these mathematic and spatial patterns suggest that other areas in the MHI with local environmental conditions similar to those between Hanaka‘ō‘ō and Papawai Points may also host higher concentrations of MHCs. Future research efforts should focus on identifying and systematically sampling locations with similar environmental conditions. Understanding the broader geographic distributions of mesophotic corals will help resource managers to effectively target ecologically important areas for conservation [76].
